# Genome Sequence of *Cronobacter sakazakii* Serogroup O:4, Sequence Type 4 Strain CDC 2009-03746, Isolated from a Fatal Case of Infantile Meningitis

**DOI:** 10.1128/genomeA.00492-15

**Published:** 2015-05-21

**Authors:** Christopher J. Grim, Gopal R. Gopinath, Karen G. Jarvis, Venugopal Sathyamoorthy, Larissa H. Trach, Hannah R. Chase, Ben D. Tall

**Affiliations:** Center for Food Safety and Applied Nutrition, U. S. Food and Drug Administration, Laurel, Maryland, USA

## Abstract

We report the draft genome sequence of a *Cronobacter sakazakii* serogroup O:4, sequence type 4 strain, CDC 2009-03746 (=NM1240=2009-06-01), isolated from a fatal case of infantile meningitis. The draft genome has a size of 4,492,904 bp and a G+C% content of 56.7.

## GENOME ANNOUNCEMENT

*Cronobacter* comprise a diverse genus of pathogens that cause life-threatening infantile infections, such as meningitis, necrotizing enterocolitis, and septicemia (1, 2). Baumbach et al. (3) recently reported the isolation of three *Cronobacter sakazakii* strains, from two infant cases, which belong to the newly described *C. sakazakii* O:4 molecular serotype (4). One of the three isolates, *C. sakazakii* strain 2009-03746 (=NM1240=2009-06-01), also belongs to sequence type 4 (ST4; http://pubmlst.org/cronobacter), which is implicated as a major cause of neonatal infections (5). To date, clinical ST4 strains which have been sequenced have been shown to belong to the Csak O:2 molecular serotype (6–8).

To further understand this isolate, whole-genome sequencing was performed on the MiSeq platform (Illumina, San Diego, CA, USA), utilizing 500 cycles of paired-end reads (Illumina). Fastq datasets were *de novo* assembled with CLC Genomics Workbench version 7.0 (CLC bio, Aarhus, Denmark). The draft genome of 2009-03746 is 4,492,904 bp, on 128 contigs (>200 bp in size) and has a G+C% content of 56.7. Genomic contigs were annotated using the RAST annotation server (9), and predicted to contain 4,170 coding sequences.

Interestingly, the genome of *C. sakazakii* 2009-03746 has a high nucleotide identity (average nucleotide identity [ANI] of 99.8%) with members of the clonal *C. sakazakii* O:2 group (10), such as *C. sakazakii* 2151 and *C. sakazakii* ES713 (GenBank accession numbers AJKT00000000.1 and AJLB00000000.1, respectively). In comparison, the ANI of 2009-03746 with another Csak O:4 isolate, *C. sakazakii* E764 (GenBank accession number AJLA00000000.1), is only 98.0%. The overall nucleotide identity with Csak O:2 genomes would be higher, except for the presence of an apparent recombination event(s) involving the 171-kb region located between tRNA-Proline-GGG and tRNA-Asparagine-GTT_1_. This region contains genes involved in extracellular polysaccharide synthesis, including colanic acid and O-antigen biosynthesis, putative galactose and mannose LPS modification operons, and the *yeh* osmoprotectant operon. Surprisingly, in *C*. *sakazakii* strain 2009-03746, the majority of this region has high nucleotide identity with that of *C. sakazakii* ATCC BAA-894, a Csak O:1 ST1 isolate, except for the Csak O:4 O-antigen region.

In terms of genome content, *C*. *sakazakii* strain 2009-03746 possesses many of the features of the species, as determined previously (11). Of note, this strain possesses genes for nine chaperone-usher fimbriae, cellulose synthesis, and sialic acid utilization operons; a 120-kb pESA3-like virulence plasmid (12); and a CRISPR element. The genome contains an additional 52-kb plasmid, homologous to plasmid pSP291-2 of *C. sakazakii* strain SP291 (GenBank accession number CP004093.1, ST4, Csak O:2) (7), which encodes copper homeostasis and heavy metal resistance operons.

Pathogen-specific virulence factors have been discovered on a variety of mobile genetic elements, such as plasmids and transposons. The “genomic plasticity” demonstrated by *C. sakazakii* strain 2009-03746 suggests ongoing genomic rearrangement, and these recombination events will undoubtedly complicate our efforts to classify these organisms into sharply delineated genomo-pathotypes.

### Nucleotide sequence accession numbers. 

This whole-genome shotgun project has been deposited at DDBJ/EMBL/GenBank under the accession number JZDO00000000. The version described in this paper is version JZDO00000000.1
